# A Modeling and Experiment Framework for the Emergency Management in AHC Transmission

**DOI:** 10.1155/2014/897532

**Published:** 2014-02-16

**Authors:** Bin Chen, Yuanzheng Ge, Laobing Zhang, Yongzheng Zhang, Ziming Zhong, Xiaocheng Liu

**Affiliations:** College of Information System and Management, National University of Defense Technology, Changsha 410073, China

## Abstract

Emergency management is crucial to finding effective ways to minimize or even eliminate the damage of emergent events, but there still exists no quantified method to study the events by computation. Statistical algorithms, such as susceptible-infected-recovered (SIR) models on epidemic transmission, ignore many details, thus always influencing the spread of emergent events. In this paper, we first propose an agent-based modeling and experiment framework to model the real world with the emergent events. The model of the real world is called artificial society, which is composed of agent model, agent activity model, and environment model, and it employs finite state automata (FSA) as its modeling paradigm. An artificial campus, on which a series of experiments are done to analyze the key factors of the acute hemorrhagic conjunctivitis (AHC) transmission, is then constructed to illustrate how our method works on the emergency management. Intervention measures and optional configurations (such as the isolation period) of them for the emergency management are also given through the evaluations in these experiments.

## 1. Introduction

In recent years, public healthy events, such as SARS [1] and H1N1 [2], happen more and more frequently. They often bring severe damage to human beings, and lots of people even lost their lives during the epidemic outbreak. It is necessary to conclude effective measures for the emergency events to alleviate the damages. Acute hemorrhagic conjunctivitis (AHC) is a quite common communicable disease in many Chinese cities. The AHC outbreaks usually last one or two month and may bring secondary attack. Isolation and activity control are the most used emergency measures to limit the epidemic. But how and when to use the measures, such as the period of isolation, still deserve deeper research.

This paper focuses on a campus-scale modeling and simulation, including the agents with statistic and geographical characters, environments with geographical distribution in a campus, and the actions and contacts of agents in social networks. High schools and universities are typical places where epidemic outbreak is easy to happen [3] since the schools are relative confined spaces with high population density, and the frequent face-to-face talk provides sufficient conditions for virus spreading. Investigations show that more than 70% of outbreak cases were found in school during influenza H1N1 in 2009 [4]. Furthermore, epidemic data from campuses is well collected and thus sufficient for the modeling. As a result, campus is a good choice for us to study the epidemic transmission.

Traditional ways for the research on epidemic transmission are used to build the SIS, SIR, and their extended models, say, SEIS, SEIRS, MSEIRS, and so forth [5]. Though these models are valid and still used in the analysis of dynamic process of epidemic, they fail to capture the micro but important factors in transmission because their functions are all statistical functions, and the spread process is not described by them. And in addition, traditional modeling ways do not consider human features (such as age and movements) which could also affect the transmission.

Agent-based modeling and simulation are an effective way to simulate the epidemic transmission in high resolution. Up to now, there are several agent-based simulation tools for epidemics, such as BioWar [6], GSAM [7], and EpiSimS [8]. BioWar is a city-scale multiagent networked model that provides simulations of 56 diseases with demographic data and diagnose process model. GASM can simulate epidemic in a global scale with more than six billion agents while considering individual behaviors. The advantages of these tools are as follows:

1. heterogeneity, the heterogeneity of agents could be easily acquired by deriving different types of agents;
2. social networks, agent is designed with multiple social relationships which will affect the dynamic characters of crowd.

However, the behaviors of agents in these tools are relatively weak due mainly to the fact that spread of diseases is not simulated by the contacts among agents but by the links in networks. In this paper, we propose a framework using agent-based modeling and simulation to build an artificial society [9]. The framework provides quantificational means for computational experiments to analyze the emergency management. In the framework, artificial society is the base conception, and finite state automata (FSA) is used to describe the agent models. Real human beings are modeled as agents, and each agent is an atomic autonomous and goal-driven unit. Microbehaviors, like movement and talking, could be simulated to induce complex macroscopic phenomenon in the agent-based artificial society. Environment is another important model unit which serves as the container for the agent movements.

AHC transformation model is divided into two parts embedded in agent and environment models, respectively. So the infections among agents could be simulated in the individual level; they are triggered when the susceptible agents are communicating with the infected agents. Furthermore, the key factors of transmission could be found through computational experiments in the study of artificial campus in epidemic situation. As a result, the most effective intervention measures could be tested repeatedly with varied experiment settings. The experiments test the best configuration of intervention measures; the quantified analysis results could be used to support the emergency management in real campus.

The paper is organized as follows.  Section 2 gives the conception of artificial society.  Section 3 proposes an artificial campus. Agent, environment, and disease models are illustrated in detail in this section. With the support of artificial campus, Section 4 gives a serial of experiments. The key factors of transmission are analyzed first, and then the quantified intervention measures are tested. In the end, the paper is concluded in Section 5.

## 2. Artificial Society

Artificial society is proposed by Wang and Lansing in 2004 [11]. It is a novel approach to solve the problems in modeling complex systems [12, 13]. The structure of artificial society and its mathematical foundation are discussed in this section.

### 2.1. How to Design an Artificial Society?

**Figure 1:**
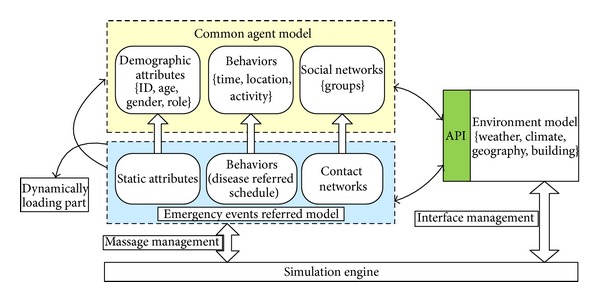
The conceptual model of artificial society [10].

With the support of agent-based modeling and simulation techniques, the conceptual model of artificial society is shown in Figure 1. It is an experimental platform for multidisciplinary modeling, analysis, and social computing. The characteristics of people are analyzed quantitatively first, and the agent models are built by statistical data. Demographic attributes, behaviors, and social networks are usually considered in agent models. Environment modes like weather and geographical information are embedded as API services to support agent modeling. But the key problem is how to model the behaviors of human because it is influenced by many factors such as social background and individual emotion. The most common approach is the statistical modeling based on the observation and analysis of real world. Statistical and observation data makes it possible to study human society. This paper discusses how to build agent model in the aspects of agent state, the state transitions, and the social networks in a campus. These models are built according to the specification of finite state automata (FSA) [14].

### 2.2. Finite State Automata: The Formalism of Models

FSA is a mathematical model of computation used to design both computer programs and sequential logic circuits. It is conceived as an abstract machine that can be in one of a finite number of states. The model could only stay in one state at a time, and the state is called current state. It can change from one state to another when initiated by a triggering event or condition; this is called a transition. A particular FSA is defined by a list of its state, and the triggering condition for each transition.

A deterministic finite state machine or acceptor deterministic finite state machine is a quintuple (*Q*, **δ**, ∑, *q*_0_, *F*), where

1. *Q* is a finite, nonempty set of states;
2. *δ* is the state-transition function (in a nondeterministic finite automaton it will be, that is, will return, a set of states);
3. ∑ is the input alphabet (a finite, nonempty set of symbols);
4. *q*_0_ is an initial state, an element of *Q*;
5. *F* is the set of final states, a (possibly empty) subset of *Q*.

## 3. Artificial Campus

According to the conceptual model of artificial society, models of artificial campus are illustrated in detail in this section.

### 3.1. Model Description

**Figure 2:**
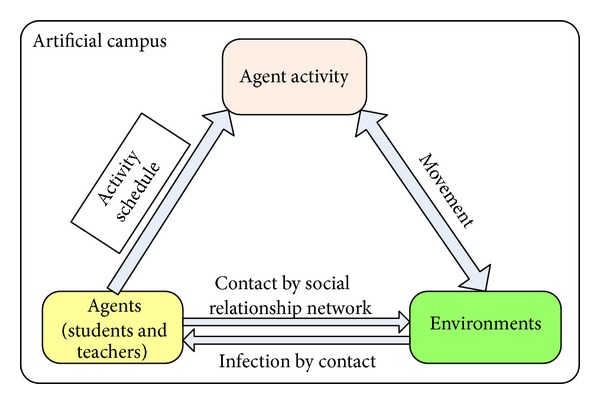
The elements of artificial campus.

As shown in Figure 2, artificial campus is composed of three fundamental elements including agent with social networks, agent activity, and environment. From the viewpoint of an agent-based system, each agent in the artificial campus represents an individual in the real world. As a result, the features of an agent model are extracted from the census figures and statistical data. Under the instruction of activity schedule, agents move from environment to another. The movements bring agent activities such as the agent contact. The contact is generated by the social relationship networks in specific environment. The disease transmission mechanism is implemented in the specific environment model. In addition, the transmission routes are determined by the contact networks constructed by social relationship networks and random networks.

### 3.2. Agent

#### 3.2.1. State Space and State Transitions

According to the specification of FSA, agent is implemented in two parts, namely, the state space and state transitions. The state space of an agent includes demographic attributes and behavior related attributes. The demographic attributes (such as age and gender) are used to model the common characteristics of people while the action related attributes (such as action and location) are used to model the student movements in the campus. More specifically, as the artificial campus is implemented for epidemic transmission, epidemic referred attributes such as immunity, health status, infected phases, infected rate, and infectivity rate are also considered in the state space of agent.

In our artificial campus, agents are grouped into students and teachers. There exist slight differences in actions and locations between the two groups. The state spaces with initialed value range of the student and teacher agents are listed in Table 1.

**Figure 3:**
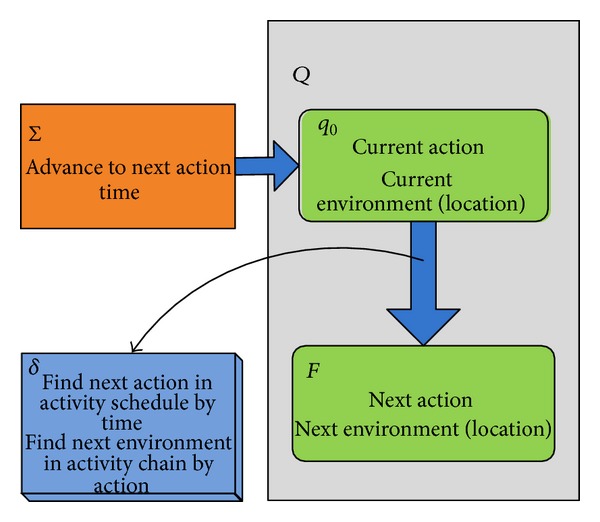
The state transitions of agent.

The state transitions of an agent include the transitions of behaviors and the transitions of disease referred attributes. The time-location-action-degree model is used to simulate action transitions as shown in Figure 3. State transitions are triggered by time. When the time is advanced to the next action time, the condition of state transitions is satisfied, and new actions and locations are computed under the instruction of activity schedule and activity chain. The transitions of disease referred attributes will be discussed later.

#### 3.2.2. Social Relationship Networks

**Figure 4:**
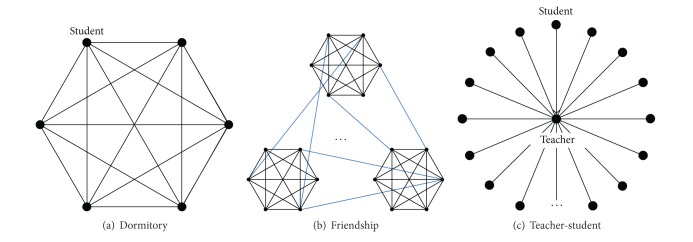
The social networks in artificial campus.

Based on the theory of complex networks, social relationship networks are built to describe the social relationships in real world. There exist many kinds of social relationships in our society. But in the artificial campus, we only consider three kinds of social relationship networks including dormitory relationship, friendship, and teacher-student relationship. Based on the dormitory distribution of universities in China, six students who have same gender are assigned to one room. Normally, students in the same dormitory have tight contacts, and the network of one dormitory is a complete graph with six vertices (see Figure 4(a)).

Friendship networks are based on dormitory networks, and all the members in one dormitory are friends of each other. However, friends of a student are not confined in dormitory members. Besides dormitory members, students can have friends in other dormitories (see Figure 4(b)). Every student has a friend list, and we complement the student's friends list by using friendship degree distribution following Poisson distribution:

(1) $$\begin{array}{c} P\left(k\right)\sim \frac{\lambda^{k}}{k!}\exp\left(-\lambda \right),\;k=0,1,2\ldots ,\;\;\lambda =8. \end{array}$$

Every student is able to get a casual friend number generated by Poison distribution. If the casual number is more than five, then select friends in the class randomly and add to his or her friend list. Contrarily, the number of friends stands at five. Teacher-student relationships are constructed of star networks (Figure 4(c)). Teacher is the centre vertex, and students are circumambient vertices. Every student has teacher-student relationship with a teacher.

#### 3.2.3. Activity Schedule

According to the state transitions of an agent, the actions of agents are determined by activity schedule. Activity schedule lists all the actions with probability in one day for agents both in normal and emergent situation. There are three types of activity schedule in artificial campus: student agent activity schedule, teacher agent activity scheduled and emergent agent schedule. For example, Table 2 gives an agent activity schedule. Upon the instruction of activity schedule, student agent changes the actions by *p*_*i*_ after state transitions. The *p*_*i*_ in the table means the action probability in the relevant period. In the duration from 08:00 to 12:00, a student agent either goes to classroom to have class or goes to library to study. The probability of class action is *p*_2_ (0.77) while the probability of study action is 1 − *p*_2_ (0.23). The probabilities in the table are set up according to the survey data of college students in China [15].

### 3.3. Environment Model

#### 3.3.1. State Space and State Transitions

In our artificial campus, environment is regarded as the container for agents to contact each other. The infection may happen during the contact if some agents are infected. The state space of environment shown in Table 3 is composed of agent population, contact activity, and environment type. In addition, the relative humidity and the air temperature are taken into account to do the infection computation.

**Figure 5:**
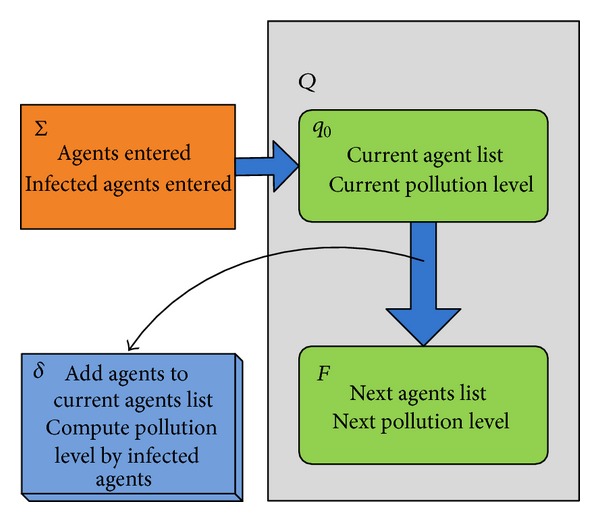
The state transitions of environments.

The transitions of environment states are triggered by agent movements. When agents entered into the environment, the agent list will be updated. According to the specification of FSA, the state transition of environment is shown in Figure 5.

#### 3.3.2. Contact Frequency and Contact Time of Agent in Environment

In the survey on AHC spreading [16], susceptible individuals will be infected by an infected individual with a probability of 43%–56% if they stay together and contact frequently (like in the household). It is because “eye-hand-eye” is the main transmission media of Acute Hemorrhagic AHC [17]. However, if a susceptible individual does not contact an infected individual too much, the infected rate is extremely low. Hence, the contact frequency is the crucial element to determine the infected rate. So, it is important to model the contact frequency and contact time of individuals.

Based on the studies on the contact behavior of human being by questionnaire survey, Edmunds et al. found that the contact frequency of individual could be fitted approximately into a normal distribution, and the mean and the standard deviation distribution are 16.8 and 8.5 [18]. So, it is possible to apply normal random variable to model the contact frequency. In our work, Box-Muller method [19] is used to generate the random variable of contact frequency, shown in the following equation:

(2) $$\begin{array}{c} F_{C}=\mu_{F}+\sigma_{F}{\left(-2\ln\left(\gamma_{1}\right)\right)}^{1/2}\cos\left(2\pi \gamma_{2}\right), \end{array}$$

in which *F*_*C*_ is the random variable of contact frequency, *μ*_*F*_ is the mean value of normal distribution, *σ*_*F*_ is the standard deviation of normal distribution, and *γ*_1_ and *γ*_2_ are the uniform random variables distributed in the interval [0, 1]. Based on (2) and survey data [15], the contact frequencies of agent *F*_contact_(*A*_*i*_) are discretized as Table 4 within the consideration of activity differences.

Similarly, the duration per contact between individuals is another key factor of AHC transmission. The duration of contact could also be modeled by the normal random variable as follows [20]:

(3) $$\begin{array}{c} T_{C}=\mu_{T}+\sigma_{T}{\left(-2\ln\left(\gamma_{1}\right)\right)}^{1/2}\cos\left(2\pi \gamma_{2}\right), \end{array}$$

in which *T*_*C*_ is the random variable of duration per contact, *μ*_*T*_ is the mean value of normal distribution, *σ*_*T*_ is the standard deviation of normal distribution, and *γ*_1_ and *γ*_2_ are the uniform random variables distributed in the interval [0, 1]. In addition, because the time spent in specific location *T*_Location_(*A*_*i*_) also follows the random distribution like (3) in our work, in order to make the duration of contact *T*_contact_(*A*_*i*_) shorter than the *T*_Location_(*A*_*i*_) the mean and standard deviation of (3) are set up as follows [20]:

(4) $$\begin{array}{c} \mu_{T}=\frac{T_{\text{Location}}}{\mu_{F}+\sigma_{F}},\\ \sigma_{T}=\frac{T_{\text{Location}}}{10\mu_{F}+10\sigma_{F}}, \end{array}$$

in which *T*_Location_ is the time of an activity in a specific location set in Table 2. *μ*_*T*_ and *σ*_*T*_ are the mean and standard deviation of contact frequency. As a result, the duration of agent *T*_contact_(*A*_*i*_) in specific locations (listed in Table 2) are also discretized in Table 4 within the consideration of activity differences.

### 3.4. Disease Transformation Model

AHC is chosen as the epidemic disease in the artificial campus [17]. The epidemiologic features of AHC include nonlethal, self-cure, short incubation period, and short time immunity after recovery. In order to simulate the epidemic transmission, the model of AHC is partitioned to three parts: AHC model, infectivity rate model of infected agent, and infected rate model in environment.

#### 3.4.1. AHC Model in Agent

**Figure 6:**
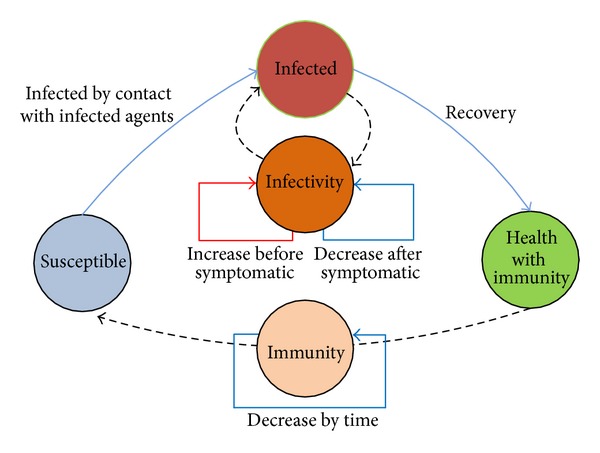
The transitions of agent health status.

AHC model simulates the states transitions of health status and the relevant actions. Referred to in SIR [5, 21], agent has three health statuses: susceptible, infected, and health with immunity. Only susceptible agent can be infected due to the contact with infected agents. After the three infected phases (incubation, symptomatic, and recovery) of AHC model, agent is healthy again. It is worth mentioning that the recovered agents can be infected iteratively when the immunity disappears gradually by time. The state transitions are illustrated in Figure 6; the immunity and infectivity of agent are also considered. The immunity is decreased by time, and the decline curve follows the exponential distribution. The infectivity of infected agent increases before symptomatic phase and decreases after symptomatic phase. The infectivity rate will be discussed in Section 3.4.5.

**Figure 7:**
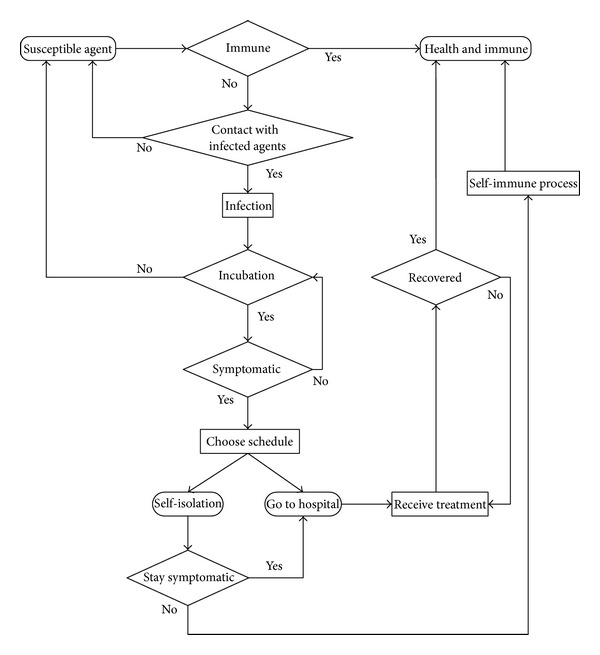
The agent infection flow.

Considering the AHC model in an agent, the work flow transitions of agent health statuses are shown in Figure 7. Based on the activity schedule and transitions rules, a susceptible agent is infected by probability only when they contact infected agents. After the infection, the agent is set in incubation phase. Not all the incubation agents will become symptomatic. Some of them turn back to susceptible, and some of them become symptomatic. The symptomatic agents change their activity schedule from normal to emergent. In the emergent case, agents go to hospital according to the treatment schedule or stay in dormitory according to isolation schedule. After the treatment in hospital or self-immune process, agents become healthy and immune of AHC. If the agents are treated in the hospital, they are not allowed to get out until recovered.

#### 3.4.2. Infectivity Rate of Infected Agent

**Figure 8:**
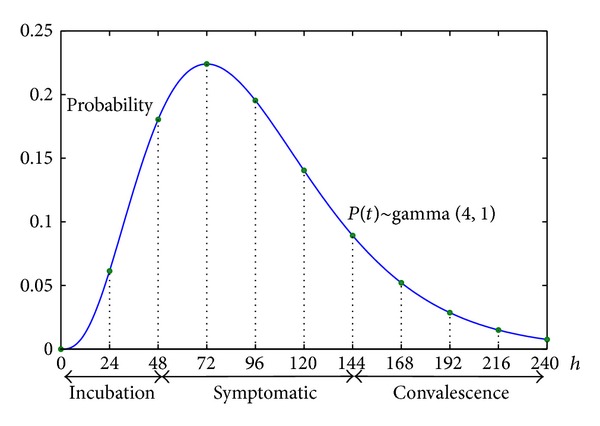
The gamma distribution of infectivity rate of an infected agent.

Infectivity decides the infection probability to susceptible agents. The infectivity rate is a function of infected phases as shown in Figure 8. Incubation period normally lasts for 0–72 hours, and, in most cases, it lasts for 0–48 hours; the symptomatic period lasts about 72 hours [21]; the convalescence period usually lasts 96 hours. According to the clinical statistic data, infectivity rate and infected duration follow the gamma distribution:

(5) $$\begin{array}{c} P_{\text{infectivity}}\left(t\right)∼\Gamma \left(4,1\right). \end{array}$$

After being infected, agents begin to have infectivity from incubation phase, and their infectivity reaches peak value 24 hours after symptoms appeared. Afterwards, infectivity declines and lasts about 168 hours after symptoms appeared.

#### 3.4.3. The Immunity of Infected Agent

**Figure 9:**
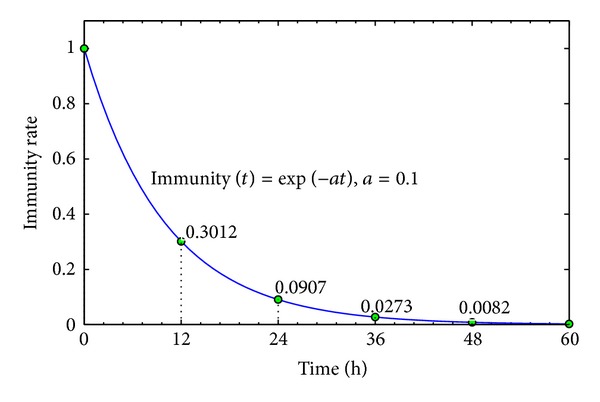
The exponential distribution of immunity since recovery of an infected agent.

According to [22], an infected individual will be immune to the AHC virus after recovery phase. But the immunity will be decreased soon in several days. So the immunity of an infected agent is approximated by exponential distribution in our work. As shown in Figure 9, the analytical expression of immunity elapsed by time is listed in (6). The immunity rate is 100% just after recovery, but it decreases to zero in 60 hours:

(6)  Immunity (t)={0t<trecoveredexp⁡(−a(t−trecovered))t>trecovered(a=0.1),

in which *t*_recovered_ is the recovered time of an infected agent.

#### 3.4.4. Relative Humidity and Air Temperature in Environment

The temperature of air and relative humidity are also important environment factors in AHC infection. These factors decide the survival of AHC virus which indicates the pollution level of environment. According to [23], in the same relative humidity of 80%, the lower the air temperature is, the lower the AHC virus survival is. Meanwhile, in the same temperature, the higher the relative humidity is, the higher the AHC virus survival is, and vice versa. Therefore, air temperature and relative humidity are quantified in several levels to model the pollution in infection computation. According to the statistical climate data of campus location [16], we fix air temperature and relative humidity on (20°C, 95.5%) in control group which is used to approximate the statistical historical data of AHC epidemic.

**Figure 10:**
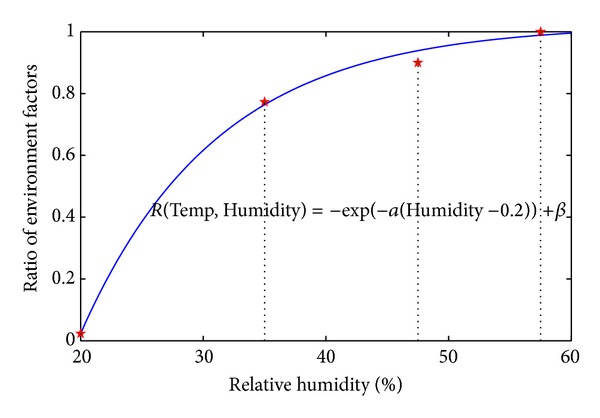
The ratio *R*(Temp, Humidity) of infection probability influenced by air temperature and relative humidity in the view of AHC virus survival.

As a result, the air temperature and relative humidity (20°C, 95.5%) are the standard settings of environment in our work. Based on the standard settings, the survival curves of AHC virus in [23] are used to model the influence on infection by air temperature and relative humidity. It means the ratio of influence of air temperature and relative humidity of 20°C and 95.5% on infection is 100% because this setting is the standard setting of air temperature and relative humidity in control group. But the ratio will be changed in other values of relative humidity. Referring to the experiment data in [23], the values of relative humidity include 20.5%, 50.5%, and 95.5% when the temperature of air is 20°C. The different virus survival curves mean the different ratios on infection. As shown in Figure 10, we generate the fitting curve of ratios with the change of values of relative humidity. The analytical expression of ratio is listed in the following equation:

(7) R(Temp,Humidity) =−exp⁡(−α(Humidity−0.2))  +β(Temp=20°C,α=4.5,β=1.0231),

in which Temp is air temperature while Humidity is relative humidity in environment.

#### 3.4.5. Infected Rate

Generally, based on the microanalysis on AHC transmission discussed before, the infection is determined by the infectivity rate, immunity rate of infected individual, contact frequency, and contact duration. Environment factors such as air temperature and relative humidity are also added to the computation of infection. In addition, macroscopic statistic data indicates that the duration of contact with infected individual has obvious influence on infected rate in AHC epidemic. Therefore, the infection is modeled in (8). The infected rate per contact is computed by infectivity, immunity, and ratio of environment factors (*E*_infection_). Environment ratio includes the ratio of air temperature, relative humidity, and the ratio of contact duration:

(8) $$\begin{array}{c} P_{\text{infected}}\left(t\right)=P_{\text{infectivity}}\left(t\right)\times \left(1-\text{Immunity}\left(t\right)\right)\times E_{\text{infection}},\\ E_{\text{infection}}=R\left(\text{Temp},\text{Humidity}\right)\times R\left(T_{\text{contact}}\right). \end{array}$$

*P*_infectivity_(*t*), Immunity(*t*), and *R*(Temp, Humidity) are discussed in detail before; the contact frequency is also listed in Table 4. It is worthy of note that the influence of duration (*R*(*T*_contact_)) acts as an adjustable parameter in our infection model to approximate the statistic history data in the next section.

## 4. Experiments

With the support of the models mentioned before, the artificial campus is implemented by the agent modeling tool OneModel. Experiments are designed to find the factors and the intervention measures to prevent the transmission of AHC.

### 4.1. OneModel

**Figure 11:**
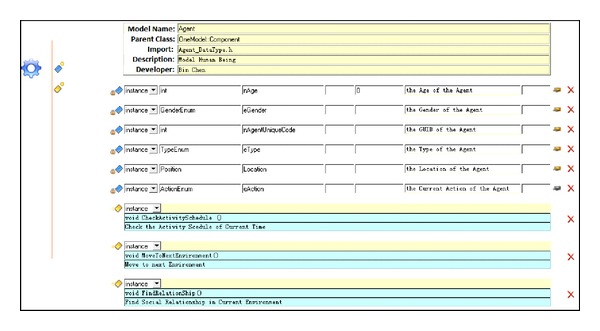
The GUI of model development in OneModel [24].

OneModel is a tool for modelers to develop agent models and generate source codes. Models of artificial campus including agent, environment, and disease models are implemented by OneModel. The GUI of model development is shown in Figure 11.

### 4.2. Map of Artificial Campus

**Figure 12:**
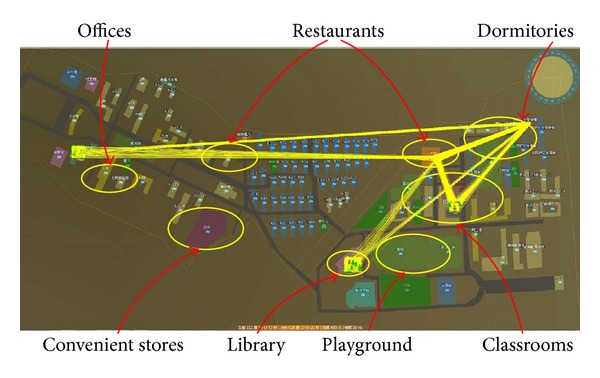
The environment map of campus.

The map of our artificial campus is shown in Figure 12; it also indicates the geographical distribution of environments. Marked by yellow ellipse, environments such as dormitories, classrooms, restaurants, offices, playground, library; and convenient stores are shown in the artificial campus. The student agents and teacher agents move from one environment to another in the map during the experiments.

### 4.3. Experiments Driven by Historical Data

The experiments are initialized by the scenario according to the historical data collected by CDC (Centers for Disease Control of China) [25]. The settings are listed in Table 5; the population of agents is 12956, including 12096 students and 860 teachers. 20 students are randomly selected to be the infected sources. As mentioned before, the ratios of duration per contact are adjustable in order to approximate the statistical historical data. The final settings of ratios are listed in Table 6. It shows that the ratio is extremely low when the contact duration is less than 5 minutes. But it increases soon when duration is larger than 10 minutes.

The effective basic reproduction number *R*_0_ is also used to show the growth rate and final size of AHC transmission. According to [26], *R*_0_ is calculated by the equation as below:

(9) $$\begin{array}{c} R_{0}=\frac{N}{C}\overset{S_{0}}{\underset{i=S_{f}+1}{\sum }}\frac{1}{i}, \end{array}$$

where *N* is agent size, *C* is the total number of infected agents in transmission, and *S*_0_ and *S*_*f*_ are the numbers of susceptible agents at the start and end of transmission, respectively. Based on (9), *R*_0_ of the historical data is 1.0315.

**Figure 13:**
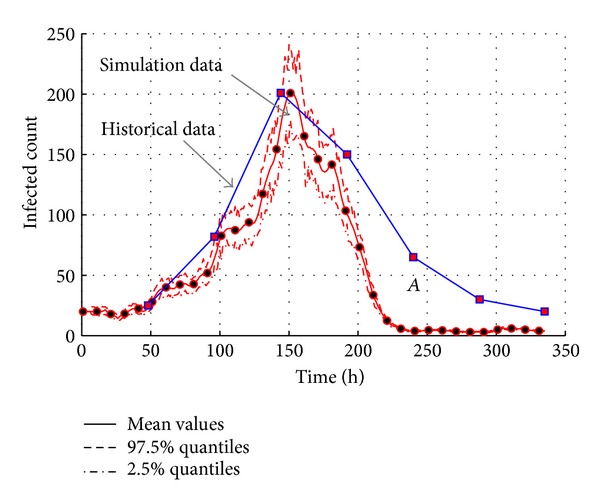
The transmission of AHC with the comparison of simulation results with the historical data.

The approximated simulation results and the statistical historical data are shown in Figure 13. As referred to in [25], the blue curve is fitted by the statistical historical data. The red curve represents the simulation data from artificial campus. It could be found that the simulated curve fits to the blue curve (historical data) in most of the time range. But the decline part of simulated curve is not consistent with the blue one, especially in the “A” area. The simulated data of infected count declines earlier than the historical data. It is because the criterions of the recovered in our models are different from the historical data. The infected agents seem to be recovered when they cannot infect others any more. But the infected individuals are considered to be healthy only when all the symptoms cannot be found in clinic. The symptomatic period always lasts for some time later than the disappearance of infectivity. This is why the blue curve of the infected declines later than the simulated curve.

It is worth mentioning that all the experiments in our work are performed 1000 times. As shown in Figures 13 to 28, experiment results are shown by curves of different styles such as solid line, dash line, and dash-and-dot line. Solid line represents the mean values of results while dash line represents the curves of 97.5% quantiles. Curves of 2.5% quantiles are represented by dash-and-dot line. All the experiments in this section are analyzed on the basis of the curves of mean values.

### 4.4. Experiments of the Sensitivity Analysis for the Factors of AHC Transmission

As summarized in [27–29], main factors of AHC transmission are the density of initial infected agents, the cleanness of environments, the agent activity, the infectivity of AHC, and the agent movements. A series of experiments are done to analyze the transmission of these factors. The initial settings of the experiments are listed in Table 7. In these experiments, the parameters such as initial infected count are directly adjusted in three cases of designated ranges to test the speed and intensity of AHC transmission. It is worthy of note that other parameters still follow the settings in control group. Compared with control group (historical data), the transmission sensitivities of these factors are analyzed in detail, respectively. The analysis results seem to be the instructions to find the intervention measures for emergency managements.

#### 4.4.1. Initial Infected Agents

**Figure 14:**
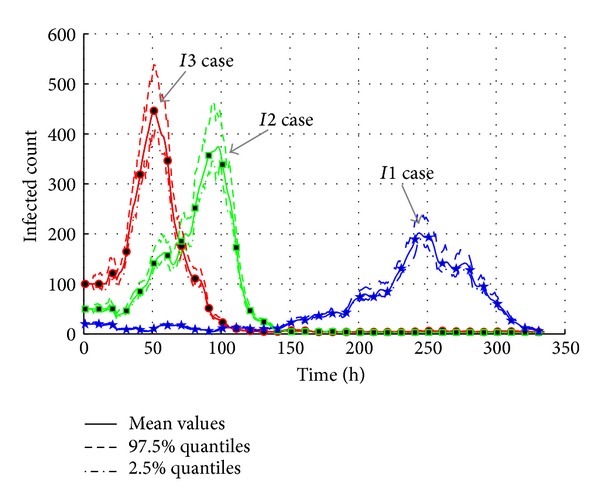
The transmission of AHC with varied densities of the sources of the infected.

When the new term begins in campus, it is possible that AHC transmission is caused by some imported cases. The initial infected agents are the imported cases of AHC. Based on the historical data, 20 imported cases are diagnosed at the beginning of the AHC transmission. So, we reset the initial infected agents to find if the “imported cases” are a key factor of AHC transmission 50 and 100 agents are set to be infected in “*I2*” and “*I3*.” The initial settings are listed in Table 8. The results are shown in Figure 14; the curve trend of “*I2*” and “*I3*” cases is similar to the control group which is initialized by “*I1*” case. The “*I2*” case represented by green curve reaches the peak point of 380 infected agents at the time of 100 h. The red curve of “*I3*” case reaches the peak point earlier than the “*I2*” case. The maximum infected count is 450 at the time of 50 h.

**Figure 15:**
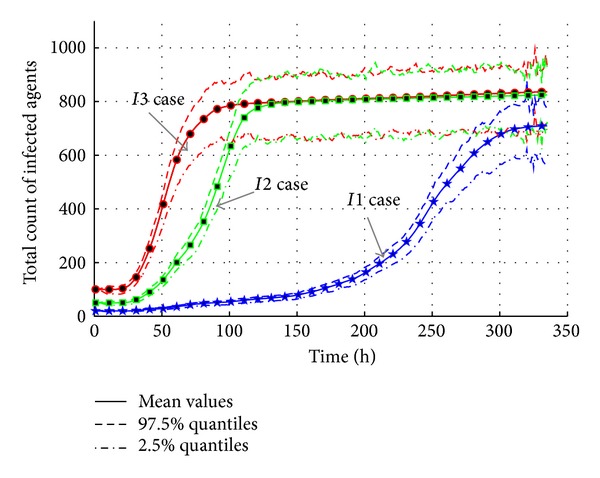
The total count of infected agents with varied densities of the sources of the infected.

Figure 15 presents the total infected count during the AHC epidemic break. The results show that the total count of infected agents is around 710 in “*I1*” case, while the count is around 830 in both “*I2*” case and “*I3*” case. The more the initial infected count is, the faster the epidemic breaks are. The interesting phenomenon is that the maximum total infected counts of “*I2*” case and “*I3*” case are similar. Based on the analysis on the initial settings of infected agents, it is found that these infected agents are limited to two classes. They have almost the same social relationship networks. Without intervention measures, most of the agents connected with the infected agents are infected. So, the different initial infected settings bring similar results.

As a result, the experiment results show that the more the number of the sources of the infected is, the quicker the peak point of the infected count will be reached, and subsequently the higher the maximum infected count is. The effective reproduction number also changes from 1.0315 in “*I1*” case to 1.0367 in “*I3*” case with a 0.5% increase. So, the number of initial infected agents is an important factor in the AHC transmission.

#### 4.4.2. Environment Cleanness

Environment cleanness is decided by the virus density, and the density is influenced by relative humidity and air temperature. As discussed in Section 3.4.4, the ratio of environment factors is modeled in infection rate. So the influence of environment cleanness can be tested by the adjustment of ratio parameters. Three designated uniform distributions of ratios are listed in Table 9.

**Figure 16:**
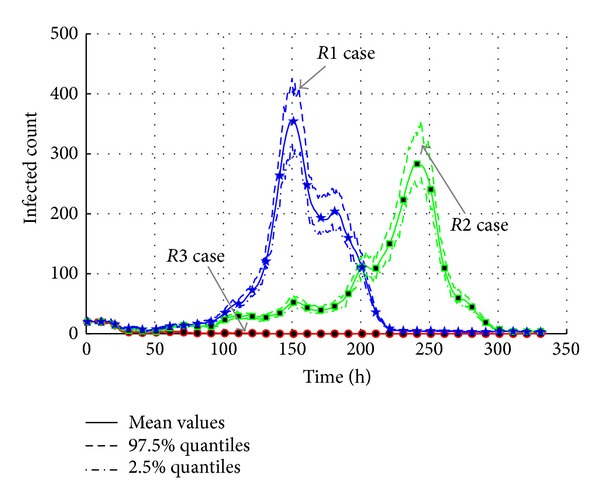
The transmission of AHC with varied cleanness levels in environments.

**Figure 17:**
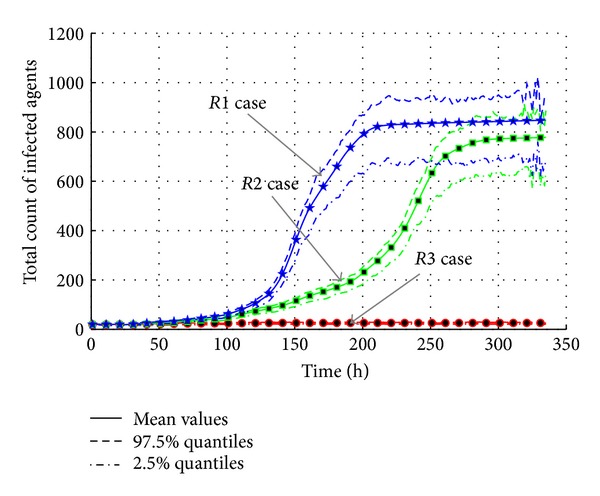
The total count of infected agents with varied cleanness parameters in environments.

The results of experiments are shown in Figures 16 and 17. In the “*R1*” case, the maximum infected agents are more than 350 with the peak time at around 150 h. The total infected count is more than 850, which is a serious epidemic break. Meanwhile, the maximum infected population of “*R2*” case is less than 300 at around 240 h. The total infected count is around 780. It is because the ratio in “*R2*” case still ranges from 0.5 to 0.8. So it is also a large-scale transmission. But in the “*R3*” case, both the maximum infected count and the total infected count are very low, so the transmission does not happen.

It is obviousl that the transmission is influenced by the cleanness of environments. The cleaner the environments are, the lower the possibility of transmission is, and the less the agents will be infected. The values of effective reproduction number testify the conclusion. *R*_0_ decreases from 1.0376 in “*R1*” case to 1.0344 in “*R2*” case, while the value 1.0019 in “*R3*” case is almost close to one. It is because the ratio in “*R3*” case is limited in *U*(0.0,0.1); it means the media of AHC transmission are cut down. Therefore, almost nobody is infected.

#### 4.4.3. Agent Contact

As the contact frequency is also a factor of the AHC transmission, agent contact is quantified to do the experiments on artificial campus. In order to test the transmission sensitiveness, contact frequency and duration are adjusted by the settings of designated normal distributions listed in Table 10. Contact model discussed in Section 3.3.2 is ignored in this experiment.

**Figure 18:**
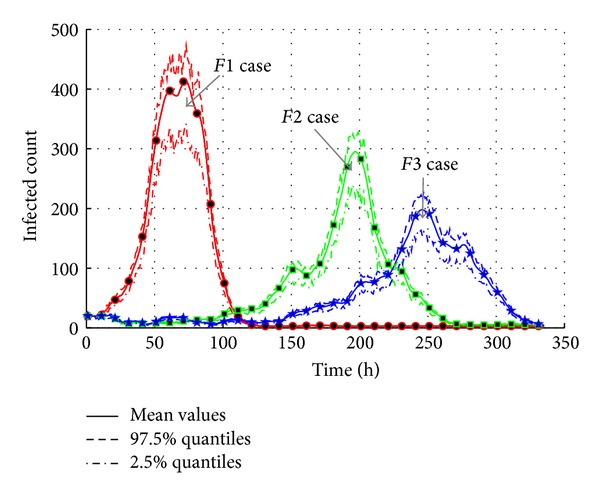
The transmission of AHC with varied contact frequencies.

As shown in Figure 18, the “*F3*” case only causes around 200 infected agents at the peak point, and the time is much later than the “*F2*” and “*F1*” cases. In “*F1*” case, the maximum infected population is almost 420 at about 70 h. It is twice as the “*F3*” case, and the second maximum infected count almost goes beyond the 400.

**Figure 19:**
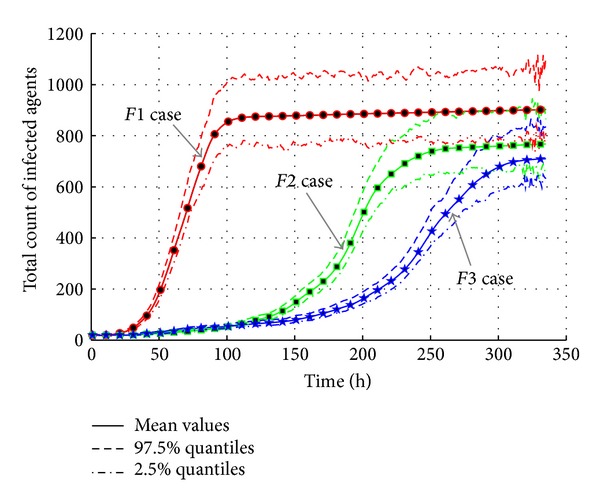
The total count of infected agents with varied contact frequencies.

Figure 19 also presents the AHC transmission by total infected count. It is obvious that contact frequency is an important factor of transmission. In “*F1*” case with high contact frequency and long contact duration, the total count of infected jumps to peak value is around 920 before 100 hours while “*F2*” and “*F3*” cases are later than 250 hours. The final infected count of “*F3*” case is 710 which is less than the “*F1*” case.

As a result, the effective reproduction number is calculated to show that the AHC transmission is sensitive to contact frequency and duration. The slight change of contact parameter brings obvious change in transmission. The effective reproduction number changes from 1.0399 in “*F1*” case to 1.0308 in “*F3*,” which means an obvious 0.9% decrease.

#### 4.4.4. Infectivity

Based on the analysis of pathological mechanism of AHC, infectivity rate could be changed to test the intensity and speed of transmission. The original normal infectivity without manually change in simulation experiments, the rate increased by 10% by manually settings in simulation experiments, the rate increased by 15% by manually settings in simulation experiments.

**Figure 20:**
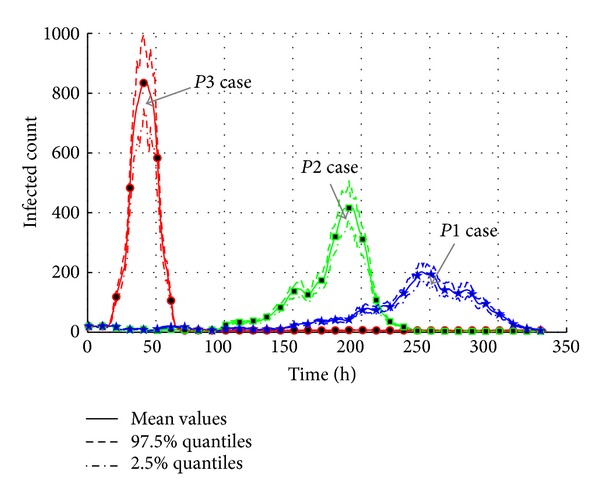
The transmission of AHC with varied infectivity rates of disease.

As shown in Figure 20, the forcible increase in infectivity rate brings the serious epidemic outbreak in short time. The differences of total infected population between three cases are not too big, but the “*P3*” case causes a rapid outbreak from 40 h to 60 h. The maximum of normal infectivity (“*P1*” case) is only 200 while the “*P3*” case is more than 800. It means the higher the infectivity is, the quicker the epidemic will be.

**Figure 21:**
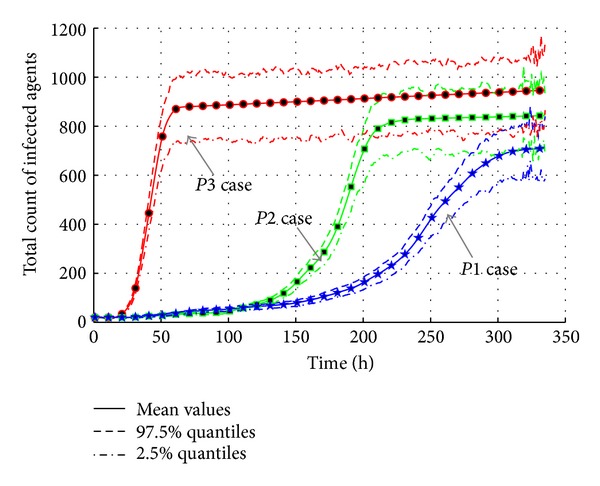
The total count of infected agents with varied infectivity rates of disease.

Figure 21 presents the total count of infected agents with different infectivity rates. The curves show that the increase of infectivity rate brings larger infected population. The “*P2*” increase case (around 850) is 140 times more than the normal infectivity case (around 710). The “*P3*” increase case (around 930) is 220 times more than the normal infectivity case (around 710). The effective reproduction number given in Table 11 also shows a rapid increase from 1.0315 in “*P1*” case to 1.0417 in “*P3*” case. So, it can be estimated that the diseases with stronger infectivity such as H1N1 and SARS could lead to a more serious epidemic disaster.

#### 4.4.5. Agents Movements

**Figure 22:**
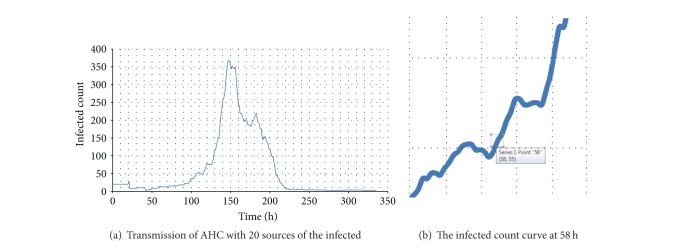
The relationship between the transmission of AHC and the agent movements.

The AHC transmission is also influenced by the agent movements to some extent.  Figure 22 gives an instance of control group. The zoomed curve around time 58 h is shown in Figure 22(b). It is easy to find that there exists a jump of infected population at 58 h (10:00 in the morning). According to the activity schedule, student agents move to the environments such as classrooms, library, and offices. The crowds of agents are formed so that the infections happen if some agents are infected. As a result, agent movements could determine the trend of transmission by activity schedule.

In summary, it is worthy of note that experiments in Section 4.4 are used to test the sensitivity of transmission factors. So, the values of parameters are adjusted without the consideration of real world. Because the epidemic experiments in real campus are really cost, and dangerous, artificial campus proposed in our work is used to do the experiments. Meanwhile, these experiments prove that the conception of artificial campus is feasible in the study of AHC transmission.

### 4.5. Emergency Intervention Measures

The epidemic outbreak will happen if no intervention measures are taken on the AHC transmission. As discussed in [30, 31], if the appropriate measures are taken when the infectious disease emerges, the transmission of the disease could be slowed down and the damage could be decreased. Therefore, it is necessary and important to design emergency intervention measures in our artificial campus.

According to the experiment results acquired in Section 4.4, it is obvious that the influence of imported cases is not as strong as agent contact and infectivity rate. For example, the extreme “*I3*” case (100 initial infected) which is five times (500%) as the control group only brings 16.9% increase of total infected population. Meanwhile, the “*P3*” case (15% increase of infectivity) brings 30.0% increase. As a result, “initial infected agents” are an important factor of AHC transmission. But its influence is not as strong as “Environment Cleanness,” “Agent contact,” and “Infectivity.” Additionally, the number of imported cases is uncontrollable in real society. So, we ignore this factor in the design of intervention measures.

As a result, the corresponding measures such as maintaining cleanliness of environments, isolation of infected agents, and control activities of agents are simulated to restrain the AHC transmission. The measures are realized by the configurations of initial parameters listed in Table 12. The analysis of the experiments on intervention measures is discussed in detail in this section.

#### 4.5.1. Maintaining the Cleanliness of Environments

**Figure 23:**
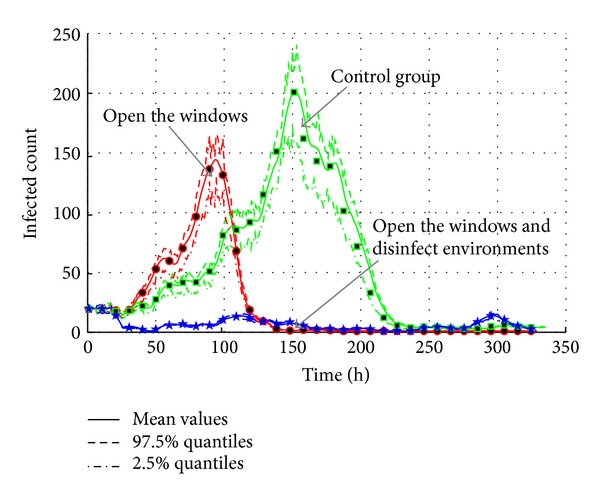
The transmission of AHC with the interventions of cleanliness maintenance.

**Figure 24:**
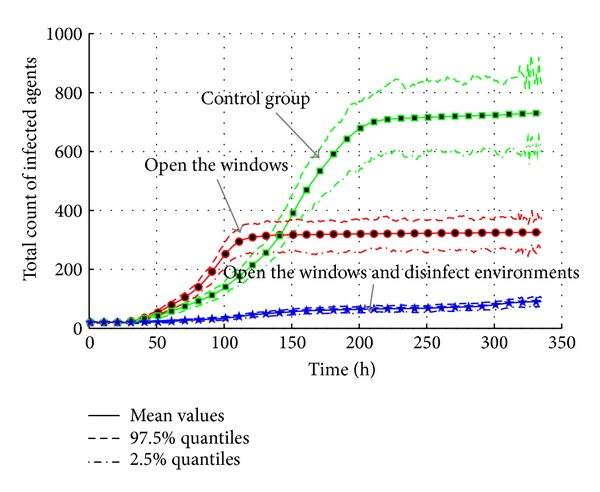
The total count of infected agents with the interventions of cleanliness maintenance.

As discussed in Section 4.4.2, the cleanliness of environment is very important in AHC transmission. So, the measures such as opening the windows and disinfecting environments are used to maintain the cleanliness of environments in experiments. We make an assumption that air temperature is not changed during the whole transmission because its period is less than 15 days. In addition, the effectiveness of the measures is represented by the decrease of relative humidity in environment. The measure of opening the window decreases average relative humidity from 95.5% to 50.5%. When the measure of disinfecting environments is added, the average relative humidity is decreased to 20.5%. The settings are listed in Table 13; the air temperature and relative humidity follow a uniform distribution. Based on the settings and the model of environment factors discussed in Section 3.4.4, the experiments results are shown in Figures 23 and 24.

As shown in Figure 23, it is obvious that these measures are effective in lowering the total infected population. Compared with control group, the survival of virus is lowered according to Section 3.4.3. Therefore, the transmission is slowed down, and the maximum infected population is only 150. When the measure of disinfecting environments is added, the probability of infectivity is set to 0. Therefore, the epidemic outbreak will not happen because the transmission media iares cut down.

From the view of total count of infected agents in Figure 24, it also shows that the intervention measures decrease the infected population rapidly. Only “Open the windows” measure decreases the total infected count from around 710 to around 340. The count of double measures is only around 90. Additionally, the effective reproduction number listed in Table 13 also shows an obvious decrease from 1.0315 of control group to 1.0044 which is very close to one. It means that the possibility of epidemic is lowered greatly. So, the measures of “Maintaining the cleanliness” are effective in the response to AHC transmission.

#### 4.5.2. Isolation of Infected Agent

According to Section 4.4.4, infectivity rate is an important factor of transmission. According to [31], isolation is often used to cut the contact with infected individuals. In the experiments, the infected agents are isolated in two ways: isolation two hours after the infection and isolation 10 hours after the infection; the settings are listed in Table 14. Isolation (*A*_*i*_) means that the infected agent *A*_*i*_ is isolated in dormitory; he or she can only contact with roommates until recovered. The settings of activity schedule in isolation are listed in Table 15.

**Figure 25:**
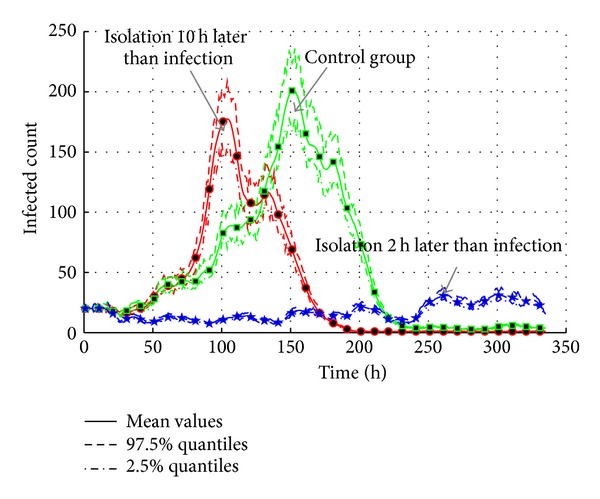
The transmission of AHC with the interventions of isolation treatments.

As shown in Figure 25, though the “10-hour” case reduces the intensity of transmission, the outbreak still causes 180 agents at the peak point, only 20 agents less than the control group. In contrast, the “2-hour” case controls the transmission of AHC; the maximum infected agents are limited; less than 40. As a result, the isolation only makes sense at the early time of transmission.

**Figure 26:**
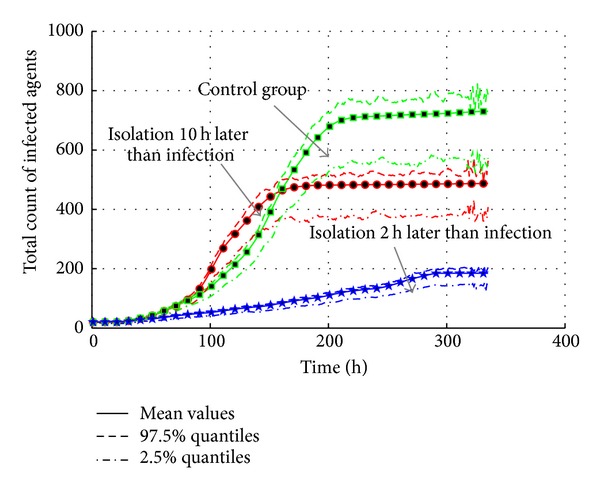
The total count of infected agents with the interventions of isolation treatments.

Figure 26 gives the total count of infected agents of three cases. Obviously, the earlier the isolation started, the less the infected population is. But the interesting phenomenon is that the “isolation 10 hours later” case gives a faster increase of infected agents from 100 h to 150 h. It is because the isolation of infected agents leads to more contact with roommates. All the roommates of infected agents are infected and isolated in dormitory. But they cannot infect other agents any more. So, the total count of infected agents stays at around 490. The effective reproduction number is 1.0213, which is 1% decrease from the control group. The “isolation 2 hours later” case shows that the timely isolation is an effective measure to cease the epidemic. Compared to the total count 710, only around 180 agents are infected in this case. The relative effective reproduction number is only 1.0082, which also means that the possibility of epidemic is very low.

#### 4.5.3. Control Activities of Agents

**Figure 27:**
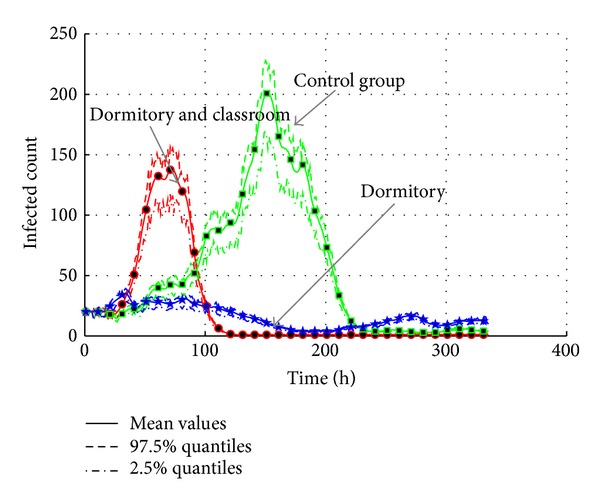
The transmission of AHC with the interventions of the control of agent activities.

**Figure 28:**
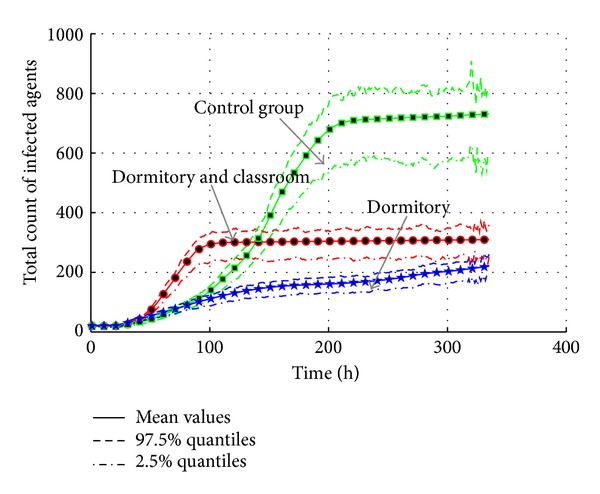
The total count of infected agents with the interventions of the control of agent activities.

According to Sections 4.4.3 and 4.4.5, the contact frequency is also an important factor of transmission. Closing some public environments can also be selected to be an intervention measure. According to Table 16, agent activities are limited in the specific locations by the new configuration in activity schedule. The activities of agents are permitted in dormitory and classroom, or only in the dormitory. The experiment results are shown in Figures 27 and 28.

Compared with the control group, the activity limitation in “dormitory and classroom” does not restrain the transmission too much. The intensity is reduced, but the outbreak is speed up. It is because the infected agents can still contact with the susceptible agents in the classroom. This type of environment still provides more chances for agents to contact the infected agents. So, the outbreak is earlier than the control group. The total infected population is reduced because the infection in other public environments is blocked by the measure. But in the “dormitory” case, infected agents could only infect the roommate agents. It will not cause a large-scale transmission.

In the view of total count of infected agents, the “Dormitory” case is more effective than the “Dormitory and Classroom” case. The effective reproduction number changes from 1.0138 to 1.0086. The possibility of epidemic is also decreased a lot because *R*_0_ is close to one. So, the control of agent activity should be more severe in order to achieve the goal of the prevention of the AHC transmission.

## 5. Conclusion

This work provides an integrated modeling and simulation framework for scientists in the field of emergency management to study complex social phenomenon. Applying demographic data, domain specific data, and survey from real world to build agent and environment model is crucial for the coherence with the real world. Social behaviors are classified by agent roles and correlative with time-geography character. Referring to the AHC transmission in a school, the artificial campus is instantiated to do the experiments to analyze epidemic transmission first. Experiment results show that agent-based model can clearly represent interaction and communication between agents and transmission processes of AHC in the campus. With varied parameter settings, the intervention measures are found to restrain the AHC transmission. The results of experiments give quantified advice on how to implement the intervention measures in real campus.

Though our method works well to some extent, the framework still has four limitations. Firstly, campus cannot replace all other environments in real world. The typical environments such as community and factory need to be constructed to support the artificial society. Secondly, the detailed data is lacked to build the more precise agent behavioral model. Thirdly, the population of the campus is only more than 10^4^. The simulation engine cannot support the city level experiments with the population of 10^8^. Fourthly, the experiment results acquired in Section 4.4 are used to test the sensitivity of transmission factors. The adjustments of parameters do not consider the real situation. So, the results seem to be the reference of intervention measures.

In order to promote the framework, lots of work will be carried out along four directions in future. The first direction is to repeat these experiments of other diseases such as H1N1 and SARS in our artificial campus. In addition, it is necessary to find the continuous curves of effective reproduction number *R*_0_ which is generated by the change of intervention parameters. *R*_0_ well reflects statistical results of transmission speed and intensity in macroview. The second direction is to extend simulation scale with multifield and multiresolution modeling. The society is much more complex than campus; other typical environments need high-resolution modeling like campus in this paper. Moreover, the resolution of models such as the individual level is not necessary in the macroartificial society. So, how to reuse the high-resolution model in a low-resolution model requires lots of coherence validation and engineering implementation. The third direction is to integrate multiple sources data into models of artificial society. With the help of source data, the models such as people contact will be more precise. The most important work is to do the data mining on the huge information from open source data such as webs in the social domain. The fourth direction is the optimizations of the simulation engine. The parallel and distributed algorithms should be added in the engine to improve the performance, so that the city level artificial society could be experimented to do the research on the emergency management.

## Figures and Tables

**Table 1:** The state space of agent.

Name of attributes	Student agent	Teacher agent
Number of population	12096	860
Age	20–23	30–50
Gender ratio (male : female)	1 : 1	1 : 1
Location	{Dormitory, classroom, library, restaurant, playground, convenience store}	{Classroom, office, restaurant, supermarket, homeland}
Action	{Sleep, class, eat, study, shopping, sports, rest}	{Sleep, class, eat, library, shopping}
Immunity/vaccine	Yes/no	Yes/no
Health status	{Susceptible, infected, health with immunity}	{Susceptible, infected, health with immunity}
Infected phases	{Incubation, symptomatic, convalescence}	{Incubation, symptomatic, convalescence}
Infected rate	*F* (age, gender, health status, activities )	*F* (age, gender, health status, activities)
Infectivity rate	*F* (infected phases)	*F* (infected phases)

**Table 2:** The activity schedule of student agent.

Duration (Δ*t*)	Activity	Location	Probability	*T*_Location_ (minute)
00:00–06:00	Sleep	Dormitory	*p*_0_ (1.00)	360

06:00–08:00	Breakfast	Dormitory/restaurant	*p*_1_ (0.68)	120
	Sports-breakfast-travel	Playground-dormitory/restaurant	1 − *p*_1_ (0.32)	

08:00–12:00	Class	Classroom	*p*_2_ (0.77)	240
	Study	Library	1 − *p*_2_ (0.23)	

12:00–14:00	Lunch	Restaurant	*p*_3_ (0.90)	120
	Lunch-shopping	Restaurant-convenience store	1 − *p*_3_ (0.10)	

14:00–18:00	Class	Classroom	*p*_4_ (0.70)	240
	Study	Library	1 − *p*_4_ (0.30)	

18:00–19:00	Dinner	Dormitory/restaurant	*p*_5_ (0.63)	60
	Sports-shopping	Playground/convenience store	*p*_6_ (0.25)	
	Study	Classroom/library	1 − (*p*_5_ + *p*_6_) (0.12)	

19:00–22:00	Rest	Dormitory	*p*_7_ (0.45)	180
	Dinner-rest	Dormitory/restaurant-dormitory	*p*_8_ (0.20)	
	Study	Classroom/library	1 − (*p*_7_ + *p*_8_) (0.35)	

22:00–24:00	Rest	Dormitory	*p*_9_ (0.72)	120
	Sleep	Dormitory	1 − *p*_9_ (0.18)	

**Table 3:** The state space of environments.

Parameters	Values
Number of environments	660
Type of environments	{Dormitory, classroom, library, restaurant, playground, convenient store}
Agent population	0–600
Relative humidity	{20.5%, 50.5%, 80.5%, 95.5%}
Air temperature	{20°C, 25°C, 33°C, 35°C}

**Table 4:** The contact frequency and duration in different locations and activities by social relationships.

Social relationship	Activity	Location	*T*_contact_(*A*_*i*_) (minute)	*F*_contact_(*A*_*i*_)
Student-student	Sleep	Dormitory	0	0
	Breakfast	Restaurant	*N*(5,2)	*N*(3,2)
	Breakfast	Dormitory	*N*(5,2)	*N*(2,1)
	Sports	Playground	*N*(2,1)	*N*(5,3)
	Class	Classroom	*N*(2,1)	*N*(2,1)
	Study	Library	*N*(2,1)	*N*(2,1)
	Lunch	Restaurant	*N*(15,8)	*N*(3,2)
	Shopping	Convenience store	*N*(20,3)	*N*(2,1)
	Dinner	Restaurant	*N*(10,8)	*N*(2,1)
	Dinner	Dormitory	*N*(15,8)	*N*(3,2)
	Rest	Dormitory	*N*(6,3)	*N*(5,2)

Teacher-student	Class	Classroom	*N*(2,1)	*N*(2,1)

**Table 5:** The initial settings of experiments by historical data.

Parameters	Value
Student count	12096
Teacher count	860
Air temperature	20°C
Relative humidity	95.5%
Initial infected	20
Basic reproduction number	1.0315

**Table 6:** The ratios of duration per contact with infected agent.

Duration of contact (minute)	Ratio of duration of max infection probability *R*(*T*_contact_)
0 < *T*_contact_(*A*_*i*_) ⩽ 2	0.05
2 < *T*_contact_(*A*_*i*_) ⩽ 5	0.15
5 < *T*_contact_(*A*_*i*_) ⩽ 10	0.28
10 < *T*_contact_(*A*_*i*_) ⩽ 20	0.65
20 < *T*_contact_(*A*_*i*_)	0.8

Effective basic Reproduction number *R*_0_	1.0315

**Table 7:** The initial settings of experiments for the sensitive analysis of AHC transmission factors.

Parameters	Value		
Student count	12096		
Teacher count	860		
Initial infected	Initial^1^	Initial^2^	Initial^3^
Ratio of relative humidity and air temperature in infection model	*R* ^1^(Temp, Humidity)	*R* ^2^(Temp, Humidity)	*R* ^3^(Temp, Humidity)
Contact frequency and contact duration	*F*_contact_^1^(*A*_*i*_), *T*_contact_^1^(*A*_*i*_)	*F*_contact_^2^(*A*_*i*_), *T*_contact_^2^(*A*_*i*_)	*F*_contact_^3^(*A*_*i*_), *T*_contact_^3^(*A*_*i*_)
Infectivity	*P*_infected_^1^(*t*)	*P*_infected_^2^(*t*)	*P*_infected_^3^(*t*)

**Table 8:** The settings of initial infected agents.

Case	Parameter values	Effective basic reproduction number *R*_0_
*I*1	Initial^1^ = 20	1.0315
*I*2	Initial^2^ = 50	1.0326
*I*3	Initial^3^ = 100	1.0367

**Table 9:** The settings of environment rate by air temperature and relative humidity.

Case	Parameter values	Effective basic reproduction number *R*_0_
*R*1	*R* ^1^(Temp, Humidity) ~ *U*(0.9, 1.0)	1.0376
*R*2	*R* ^2^(Temp, Humidity) ~ *U*(0.5, 0.8)	1.0344
*R*3	*R* ^3^(Temp, Humidity) ~ *U*(0.0, 0.1)	1.0019

**Table 10:** The settings of contact frequency and duration.

Case	Parameter values	Effective basic reproduction number *R*_0_
*F1 *	*F*_contact_^1^(*A*_*i*_) ~ *N*(5,2), *T*_contact_^1^(*A*_*i*_) ~ *N*(3,5)	1.0399
*F2 *	*F*_contact_^2^(*A*_*i*_) ~ *N*(1,2), *T*_contact_^2^(*A*_*i*_) ~ *N*(2,3)	1.0355
*F3 *	*F*_contact_^3^(*A*_*i*_) ~ *N*(0,1), *T*_contact_^3^(*A*_*i*_) ~ *N*(0,1)	1.0308

**Table 11:** The settings of infectivity rate.

Case	Parameter value	Effective basic reproduction number *R*_0_
*P*1	*P*_infectivity_^3^(*t*) = *P*_infectivity_(*t*)	1.0315
*P*2	*P*_infectivity_^2^(*t*) = *P*_infectivity_(*t*) (1 + 10%)	1.0381
*P*3	*P*_infectivity_^1^(*t*) = *P*_infectivity_(*t*) (1 + 15%)	1.0417

**Table 12:** The settings of experiments for emergency intervention measures.

Parameters	Value		
Student count	12096		
Teacher count	860		
Initial infected	20		
Immunity	Immunity(*t*)		
Infectivity	*P*_Infected_(*t*)		
Air temperature	20°C		
Relative humidity	95.5%	50.5%	20.5%
Isolation of infected	Null	2 hours after infection	10 hours after infection
Activity control	Null	Dormitory and classroom allowed	Only dormitory allowed

**Table 13:** The settings of environment cleanliness.

Intervention measure	Setting	Effective basic reproduction number *R*_0_
Control group	Temp ~ *U* (20 ± 5°C), Humidity ~ *U* (95 ± 5%)	1.0315
Open the windows	Temp ~ *U* (20 ± 5°C), Humidity ~ *U* (50 ± 5%)	1.0150
Open the windows and disinfect environments	Temp ~ *U* (20 ± 5°C), Humidity ~ *U* (20 ± 5%)	1.0044

**Table 14:** The settings of isolation of agents.

Intervention measure	Setting	Effective basic reproduction number *R*_0_
Control group	No isolation	1.0315
Isolation 10 h later than infection	Isolation(*A*_*i*_), *t*_infected_ + 600 min < *t* < *t*_recovered_	1.0213
Isolation 2 h later than infection	Isolation(*A*_*i*_), *t*_infected_ + 120 min < *t* < *t*_recovered_	1.0082

**Table 15:** The activity and location of infected agents in isolation.

Activity	Location	*T*_contact_(*A*_*i*_)	*F*_contact_(*A*_*i*_)
Sleep	Dormitory	0	0
Breakfast	Dormitory	*N*(5,2)	*N*(2,1)
Lunch	Dormitory	*N*(10,8)	*N*(3,2)
Dinner	Dormitory	*N*(10,8)	*N*(3,2)
Rest	Dormitory	*N*(6,3)	*N*(5,2)

**Table 16:** The settings of control activities of agents.

Intervention measure	Activity	Location	*T*_contact_(*A*_*i*_)	*F*_contact_(*A*_*i*_)	Effective basic reproduction number *R*_0_
Dormitory and classroom	Sleep	Dormitory	0	0	1.0138
	Breakfast	Dormitory	*N*(5,2)	*N*(2,1)	
	Class	Classroom	*N*(2,1)	*N*(2,1)	
	Lunch	Dormitory	*N*(10,8)	*N*(3,2)	
	Dinner	Dormitory	*N*(10,8)	*N*(3,2)	
	Rest	Dormitory	*N*(6,3)	*N*(5,2)	

Dormitory only	Sleep	Dormitory	0	0	1.0086
	Breakfast	Dormitory	*N*(5,2)	*N*(2,1)	
	Lunch	Dormitory	*N*(10,8)	*N*(3,2)	
	Dinner	Dormitory	*N*(10,8)	*N*(3,2)	
	Rest	Dormitory	*N*(6,3)	*N*(5,2)	
